# Predicting the invasiveness of pulmonary adenocarcinoma using intratumoral and peritumoral radiomics features

**DOI:** 10.3389/fmed.2025.1541682

**Published:** 2025-05-21

**Authors:** Jingjing Hong, Liyang Yang, Jiekun Huo, Guoci Huang, Bowen Shan, Tingting Cai, Lianlian Zhang, Weikang Huang, Ge Wen

**Affiliations:** Nanfang Hospital, Southern Medical University, Guangzhou, China

**Keywords:** radiomic features, pulmonary adenocarcinoma, invasiveness, intratumoral, peritumoral

## Abstract

**Objective:**

To evaluate the predictive value of CT radiomics features within and surrounding tumors in determining the invasiveness of primary solitary nodular pulmonary adenocarcinoma.

**Methods:**

This retrospective study analyzed 107 patients with pathologically confirmed nodular pulmonary adenocarcinoma who underwent conventional non-enhanced CT Scans in our hospital from 2019 to 2023. Patients were categorized as non-invasive or invasive based on pathology findings. Clinical and imaging data from both groups were collected and compared, and logistic regression was used to independent factors associated with invasiveness. Radiologists manually outlined 3-dimensional regions of intratumoral and peritumoral areas to extract radiomics features, creating separate intratumor, peritumor, and combined intra-peritumor radiomics models. Radiomics models were trained using LASSO with 10-fold cross-validation in training dataset. Additionally, integrated models combining radiomics with clinical data were developed: intratumor-clinical, peritumor-clinical, and an intra-peri-clinical models.

**Results:**

Of the 107 patients, 73 were in the non-invasive group (mean age 49.73 ± 13.92, 22 males) and 34 were in the invasive group (mean age 57.53 ± 12, 14 males). The clinical model identified average nodule diameter and vascular type as independent risk factors for invasiveness (both *p* < 0.025). The combined intra-peri-clinical model demonstrated superior predictive performance compared to other models, with an AUC of 0.93, sensitivity of 0.91, and specificity of 0.86.

**Conclusion:**

The combined model incorporating intratumor and peritumor radiomics features with clinical data showed significant value in predicting the invasiveness of nodular pulmonary adenocarcinoma, aiding in the precise selection of surgical methods.

## Highlights

- CT Radiomics Predict Invasion in Lung Adenocarcinoma.
- Combined Model Outperforms in Predictive Accuracy.
- Clinical Data Enhances Radiomics Prediction.

## 1 Introduction

Lung cancer remains a formidable public human health challenge, representing one of the most aggressive malignancies (1). Among its various histological subtypes, pulmonary adenocarcinoma, the most common histological subtype, in early-stage lung cancer cases (2), encompassing a spectrum of precursor glandular lesions (PGL), including atypical adenomatous hyperplasia (AAH), adenocarcinoma *in situ* (AIS), minimally invasive adenocarcinoma (MIA), and invasive adenocarcinoma (IAC) (3, 4), as defined by the 2021 WHO Classification of Thoracic Tumors (5th edition). These entities typically present as sub-solid lung nodules on CT imaging, featuring both pure ground-glass and occasionally solid nodules appearance (3, 4).

The progression from AAH through AIS and MIA to IAC represents a continuum of increasing malignancy (3). Management strategies differ significantly across this spectrum; AIS/MIA typically necessitates wedge or segmental resection to conserve pulmonary function, whereas IAC usually necessitates lobectomy with lymph node dissection.

Clinical studies, including a multicenter prospective trial by Travis et al., have demonstrated that intraoperative frozen section-guided sublobar resection achieves near 100% 5-year recurrence-free survival in patients with adenocarcinoma *in situ* (AIS) and minimally invasive adenocarcinoma (MIA), significantly outperforming outcomes in invasive adenocarcinoma (IAC) (sensitivity 94.3%, specificity 89.6%) (5). Accurately differentiating IAC from less invasive forms preoperatively is crucial for tailoring effective therapeutic strategies, although this remains challenging when relying solely on CT imaging (6, 7).

Radiomics, which involves extracting and analyzing quantitative features from medical images using machine learning techniques, reveals tumor characteristics like shape, texture, and intensity, and offers a promising solution by enhancing diagnostic accuracy and predictive capabilities (8–17). Recent advancements in radiomics, particularly within the domain of pulmonary adenocarcinoma, have focused on utilizing high-resolution imaging and sophisticated algorithms to elucidate the microstructural and biological characteristics of tumors. In recent years, research on radiomics has encompassed several aspects. For instance, the diagnostic efficacy of differentiating histological subtypes of lung adenocarcinoma using radiomics or deep learning networks has been reported to range from 73.0 to 91% (13–15). Additionally, studies have explored the prognostic and therapeutic implications of radiomics in lung adenocarcinoma. For example, a PET/CT-based radiomics model demonstrated good performance in predicting intermediate-high risk growth patterns in early invasive adenocarcinoma (IAC), providing a valuable method for clinical management and personalized treatment (16). Furthermore, research has also focused on identifying the epidermal growth factor receptor (EGFR) gene mutation status in lung adenocarcinoma, which is crucial for determining the use of EGFR-tyrosine kinase inhibitors (EGFR-TKIs) and thus beneficial for personalized patient care (17). While radiomics has been widely explored in pulmonary adenocarcinoma, our work uniquely integrates both intratumoral and peritumoral radiomics features with clinical data to predict invasiveness. This dual-region approach, coupled with clinical factors, offers a novel, holistic framework for preoperative decision-making, addressing a gap highlighted in recent literature.

## 2 Information and methods

### 2.1 General information

The retrospective analysis involved clinical and imaging data obtained from 107 patients with lung nodules who underwent preoperative conventional non-enhanced CT scanning at our hospital between January 2020 and December 2023. Patients were categorized into two groups based on pathological findings: 73 cases in the non-invasive group (comprising AAH, AIS and MIA), and 34 cases in the invasive group (IAC). The inclusion criteria were: lung nodules with a diameter of less than 30 mm; preoperatively isolated lung nodules confirmed through routine plain CT examination; CT images of diagnosable quality without significant artifacts; availability of complete pathological results after lung resection.

The exclusion Criteria included: incomplete CT imaging and clinical history data; nodule diameter exceeding 30 mm, and poor image quality hindering diagnostic assessment.

This retrospective study used anonymized data, ensuring compliance with patient privacy regulations. Therefore, it was deemed exempt from the requirement for informed consent and ethical review, as per the guidelines of our institutional ethical review board.

### 2.2 Imaging procedures

All chest CT scans were conducted utilizing a 256-row CT scanner (Revolution Apex, GE Healthcare). Before scanning, patients received respiratory training to ensure optimal breath-holding at the end of inspiration. Scans covered the entire lung volume, from apex to base. The following scan protocol was set: tube voltage of 120 kV, noise index of 11HU, automatic tube current modulation, 5 mm scanning slice thickness and spacing, image reconstruction utilizing adaptive statistical iterative-Veo (ASIR-V) at 60% weighting with a slice thickness of 0.625 mm, and the lung kernel for reconstruction. The image display parameters were set to a window width of 1,600 HU and a window position of −550 HU.

### 2.3 CT image characterization

Two experienced attending radiologists (XXX and XXX) specializing in diagnostic chest imaging conducted blinded reviews of the CT images. The final imaging characteristics were confirmed through consensus and discussion, including nodule types (pure ground-glass density nodules, partially solid nodules, and solid nodules), lesion locations (upper, middle, and lower lobes of the right lung; upper and lower lobes of the left lung), average nodule diameter, nodule shape (round, irregular), Lobulation sign, nodule edges (smooth, irregular), nodule boundaries (clear, indistinct), presence of burr sign, pleural pulling sign, emphysema, air bronchus sign, air bubble sign andvascular type (low-grade indicating absence or normal morphology of vascular routes, high-grade indicating abnormal morphology or distortion).

**Figure 1:**
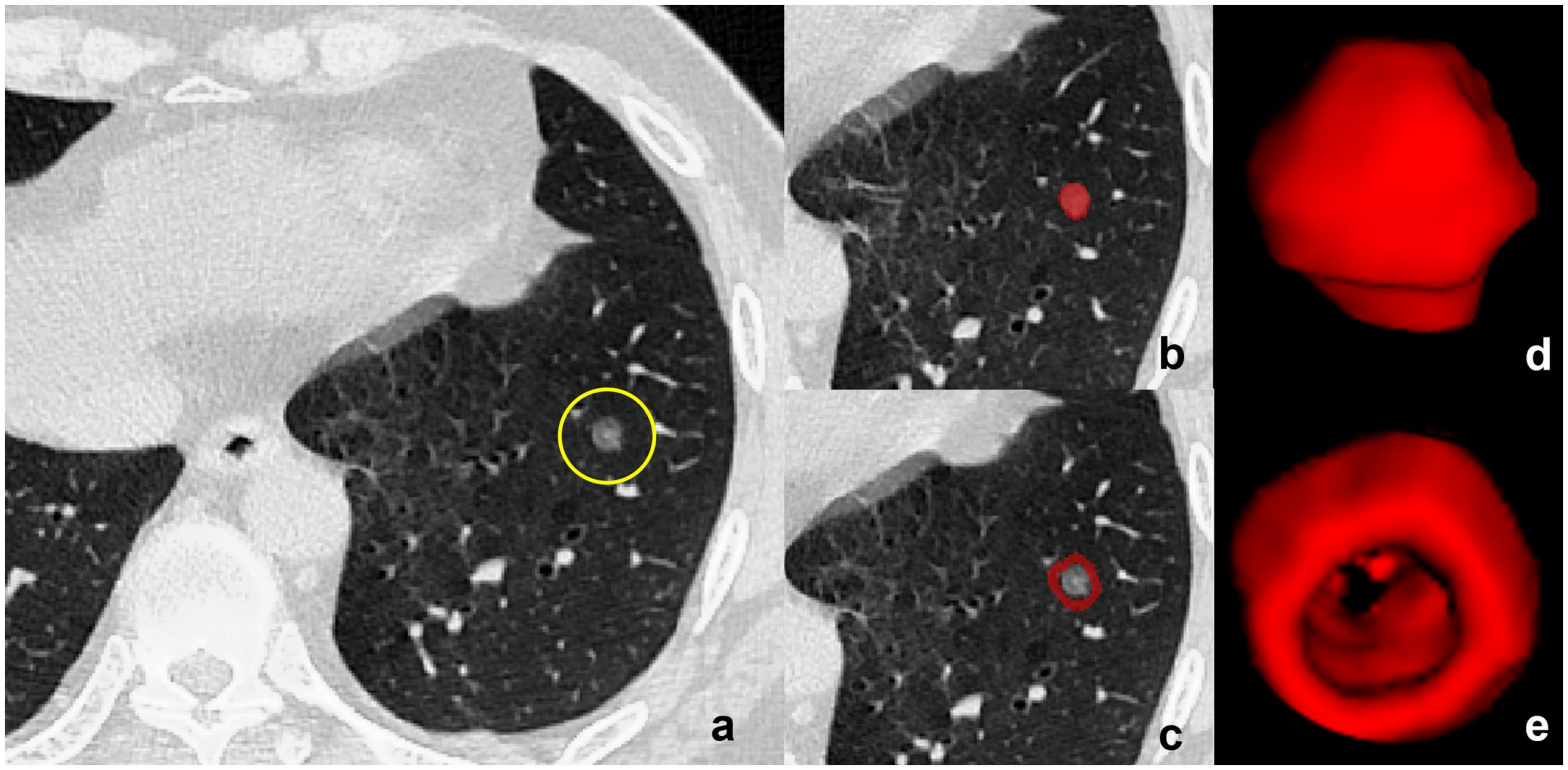
Delineation of Tumor and Peritumoral Regions. **(a)** CT image of a subsolid lesion (encircled in yellow) with pathological confirmation of invasive pulmonary adenocarcinoma. **(b)** The region of interest (ROI) within the tumor is manually demarcated layer by layer, adhering to the inner boundary of the neoplastic mass. **(c)** Three-dimensional (3D) model of the intratumoral volume rendered in ITK-SNAP, showcasing the volumetric configuration of the tumor’s interior. **(d)** The peritumoral region is displayed based on an automated segmentation program with dilation, ensuring the exclusion of all proximal large blood vessels to mitigate potential analytical interference. **(e)** Construction of the peritumoral volume’s 3D model within ITK-SNAP, illustrating the morphological and dimensional attributes of the area circumambient to the tumor, essential for an exhaustive radiomic assessment.

A senior radiologist (XXX) with over 5 years of clinical expertise conducted precise manual segmentation of the lesion’s region of interest (ROI) based on scanned breast CT images, utilizing ITK-SNAP 3.8.0 software (refer to Figure 1). During segmentation, meticulous care was taken to avoid neighboring large blood vessels, bronchial tubes, and skeletal structures, thereby minimizing interference from anatomical structures in defining the lesion region. Subsequently, the ROIs derived from segmentation underwent thorough layer-by-layer correction to ensure accuracy.

Automated boundary expansion was then applied to the corrected ROIs using scripts written in the R programming language, expanding outward in 3 mm increments. This delineated the tumor’s peripheral region, laying the groundwork for subsequent radiomic feature analysis. Following image normalization and resampling, PyRadiomics 3.0.1 software extracted radiomics features from all ROIs, encompassing first-order, shape, texture, and higher-order statistical features.

All extracted features underwent normalization. Samples were randomly partitioned into training and test sets at a ratio of 7:3. Within the training set, sequential analyses including independent sample *t*-test, K-Best algorithm ANOVA, and recursive feature elimination were conducted to identify key features. Subsequently, a regression model employing the least absolute shrinkage and selection operator (LASSO) was applied for 10-fold cross-validation on training sets, facilitating the screening of optimal features and model training. The resulting radiomics score (Rad-score) was then computed.

### 2.4 Statistical analysis

Statistical analyses were conducted using SPSS 25.0 and Python 3.7 software. Count data were presented as frequencies, and differences between groups were assessed using the χ^2^-test. Measurement data were expressed as mean ± standard deviation, and differences between groups were analyzed using independent samples t-test. Subsequently, univariate and multivariate logistic regression were utilized to obtain the independent factors associated with infiltrative nature, and the clinical model was constructed by utilizing those independent factors.

LASSO regression was employed to establish three imaging models: the intratumor model, the peritumor model, and the intra-peritumor model, focusing on intratumoral, peritumoral, and combined intratumoral and peritumoral features, respectively. Additionally, logistic regression was utilized to establish the intratumor-clinical model, peritumor-clinical model, and intra-peritumor model based on clinical imaging, intratumor, and peritumor features.

Subsequently, logistic regression was applied to develop an intra-peri-clinical model by integrating key clinical imaging features with intratumor and peritumor radiomics scores. Receiver operating characteristic (ROC) curves were plotted for each model, and the corresponding area under the curve (AUC) was calculated to assess the models’ effectiveness in evaluating the invasiveness of pulmonary adenocarcinoma. A nomogram was used to visualize interrelationships between variables in the intra-peri-clinical model. Additionally, calibration curves were plotted to evaluate the calibration performance of the model, while decision curve analysis (DCA) was employed to assess its clinical benefit. A significance level of *p* < 0.05 was considered statistically significant.

## 3 Results

### 3.1 Clinical data and CT image characterization

According to pathological findings, 107 eligible patients were divided into a group without invasiveness - 22 males and 51 females with a mean age of (49.73 ± 13.92) years, and an invasive group with 14 males and 20 females with a mean age of (57.53 ± 12) years. The between-group difference in sex and age was statistically significant (*p* < 0.001 and 0.006, respectively).

The parameters between the two groups including nodule type, lesions location, the average nodules diameter, nodule shape, nodule edge, nodule boundary, burr sign, pleural pulling sign, emphysema background, air bronchus sign, air bubble sign, vascular type, were statistically significant (all *p* < 0.05, Table 1).

**Table 1:** Parameters for comparison of clinical and imaging characteristics between the two groups.

Parameter	Non-invasive group (*n* = 73)	Invasive group (*n* = 34)	t/χ^2^	*P*
Age/years	49.73 ± 13.92	57.53 ± 12	2.816	0.006
Gender/case			11.449	<0.001
Males	22	14		
Females	51	20		
Nodule type/case			22.51	<0.001
Pure ground-glass nodules	44	7		
Pure ground-glass nodules	25	18		
Solid nodules	4	9		
Location of the lesion			15.477	0.004
Right lung suprakane	23	14		
Middle lobe of the right lung	9	5		
Right lower lobe	15	5		
Left upper lobe	15	5		
Left lower lobe	11	5		
Average diameter of nodules	7.5 (6, 9.5)	11.75 (9, 16.5)	5.421	<0.001
Shape			4.944	0.026
Round, quasi-circular	55	10		
Irregular shapes	18	24		
Lobulation sign	29	31	1.579	0.209
Edge			74.03	<0.001
Irregular	66	32		
Smooth	7	2		
Boundary/case			32.53	<0.001
Clear	59	24		
Indistinct	14	10		
Burr sign/case	7	21	24.31	<0.001
Pleural traction sign/case	21	21	4.944	0.026
Emphysema background/case	6	8	58.33	<0.001
Air bronchial signs/cases	7	14	39.49	<0.001
Air bubble sign/case	17	8	30.36	<0.001
Vascular type/case			6.81	0.009
Low level	61	6		
High level	12	28		

Univariate and multivariate analyses revealed that average nodule diameter and vascular type were independent risk factors for pulmonary adenocarcinoma invasiveness (both *p* = 0.025 and 0.002, respectively), as presented in Table 2.

**Table 2:** Logistic regression analysis revealed independent predictors associated with the aggressiveness of adenocarcinoma.

Variable	Univariate analysis			Multivariate analysis				
	β	OR	*p*-value	β	SE	Wald χ^2^	*P*-value	OR (95%CI)
Age/years	0.045	1.046	0.008	0.029	0.031	0.913	0.339	1.03 (0.97,1.093)
Gender/case	−0.48	0.616	0.262					
Males								
Females								
Nodule type/case	1.367	3.924	<0.001	−0.15	0.564	0.071	0.79	0.861 (0.285,2.599)
Pure ground-glass nodules								
Partial solid nodules								
Solid nodules								
Lesion location/case	−0.13	0.877	0.358					
Right lung suprakane								
Middle lobe of the right lung								
Right lower lobe								
Left upper lobe								
Left lower lobe								
Average diameter of nodules	0.386	1.471	<0.001	0.258	0.115	5.037	0.025	1.294 (1.033,1.621)
shape/case	−1.99	0.136	<0.001	−0.46	0.697	0.444	0.505	0.629 (0.16,2.463)
Round, quasi-circular								
Irregular								
Edge/case	0.529	1.697	0.524					
Irregular								
Smooth								
Boundary/case	−0.56	0.569	0.24					
Clear								
indistinct								
Burr sign/case	2.723	15,231	<0.001	0.494	0.87	0.322	0.57	1.639 (0.298,9.024)
Pleural traction sign/case	1.386	4	0.002	0.075	0.668	0.013	0.91	1.078 (0.291,3.997)
Emphysema background/case	1.234	3.436	0.036	−0.57	1.084	0.273	0.601	0.567 (0.068,4.746)
Air bronchial signs/cases	1.887	6.6	<0.001	−0.02	0.853	0.001	0.985	0.984 (0.185,5.232)
Air bubble sign/case	0.013	1.014	0.978					
Vascular type/case	3.166	23.722	<0.001	2.355	0.751	9.833	0.002	10.537 (2.418,45.91)
Low level								
High level								

The clinical model alone attained an AUC of 0.89 in training and 0.91 in testing, with a sensitivity of 0.74 and specificity of 0.92 in training, and a perfect sensitivity of 1.00 with a specificity of 0.86 in testing.

### 3.2 Radiomics feature selection

Following regression modeling using the LASSO algorithm, the study identified specific features from intratumor, peritumor, and peritumor-peritumor models.

For the intratumor model, five features were selected: original_gIrlm_LongRunLowGrayLevelEmphasis, original_glcm_MCC, original_glcm_MaximumProbability, original_glszm_SizeZoneNonUniformity, and original_ngtdm_Coarseness. For the peritumor model, four features were identified: original_ngtdm_Strength, original_glcm_InverseVariance, original_firstorder_Median, and original_ngtdm_Coarseness.

**Figure 2:**
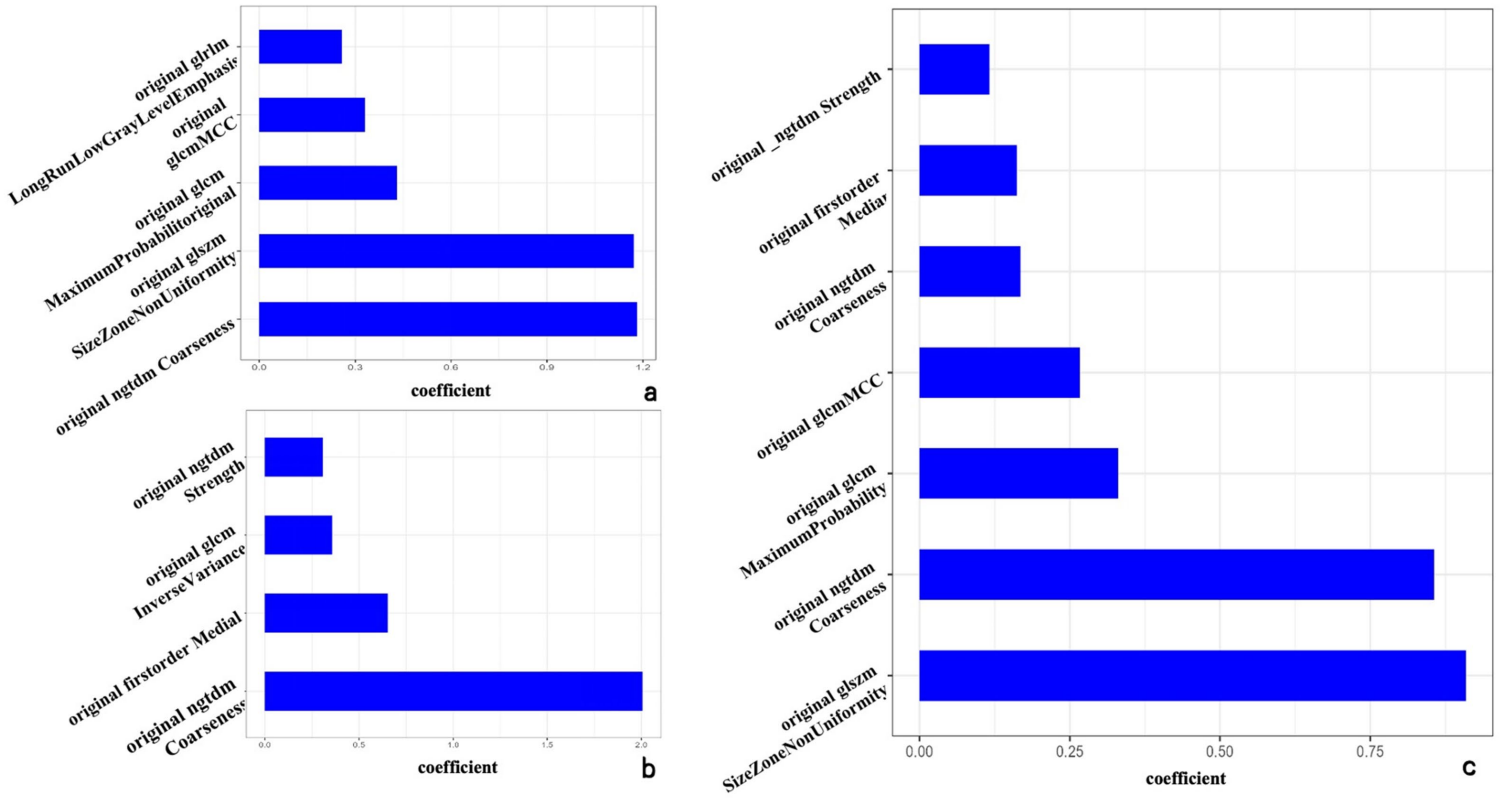
The optimal radiomics characteristics and corresponding coefficients based on the intratumor model **(a)**, peritumor model **(b)**, and intra-peritumor model **(c)**.

Additionally, seven features were selected from the intra-peritumor model: original_ngtdm_Strength, original_firstorder_Median, original_ngtdm_Coarseness.1, original_glcm_MCC, original_glcm_MaximumProbability, original_ngtdm_Coarseness, and original_glszm_SizeZoneNonUniformity (refer to Figure 2).

The Rad-score was calculated as the weighted sum of selected features and their corresponding coefficients for the intra-peritumoral radiomic model.

### 3.3 Radiomics model evaluation

The intra-peritumor model exhibited performance comparable with the intratumor model, with both achieving an AUC of 0.92 in the training and test sets. The intra-peritumor model achieved a sensitivity of 0.89 and specificity of 0.86 in training, while in the test set, sensitivity remained at 0.89, and specificity slightly decreased to 0.85. Similarly, the intratumor model exhibited a sensitivity of 0.89 and specificity of 0.81 in training, with test set values of 0.84 and 0.85, respectively.

The peritumor model showed an AUC of 0.89 in training and 0.90 in testing, with relatively lower sensitivity (0.67 in training) but a sensitivity of 1.00 in the test set. However, its specificity declined from 0.95 in training to 0.69 in testing.

The intratumor-clinical and peritumor-clinical models also exhibited strong predictive capabilities, both yielding an AUC of 0.95 in training and 0.93 and 0.92 in testing, respectively. The intratumor-clinical model achieved a sensitivity of 0.73 and specificity of 0.96 in training, improving to 1.00 sensitivity and 0.83 specificity in testing. Similarly, the peritumor-clinical model reported a sensitivity of 0.69 and specificity of 0.98 in training, while in the test set, it reached 1.00 sensitivity and 0.86 specificity.

**Figure 3:**
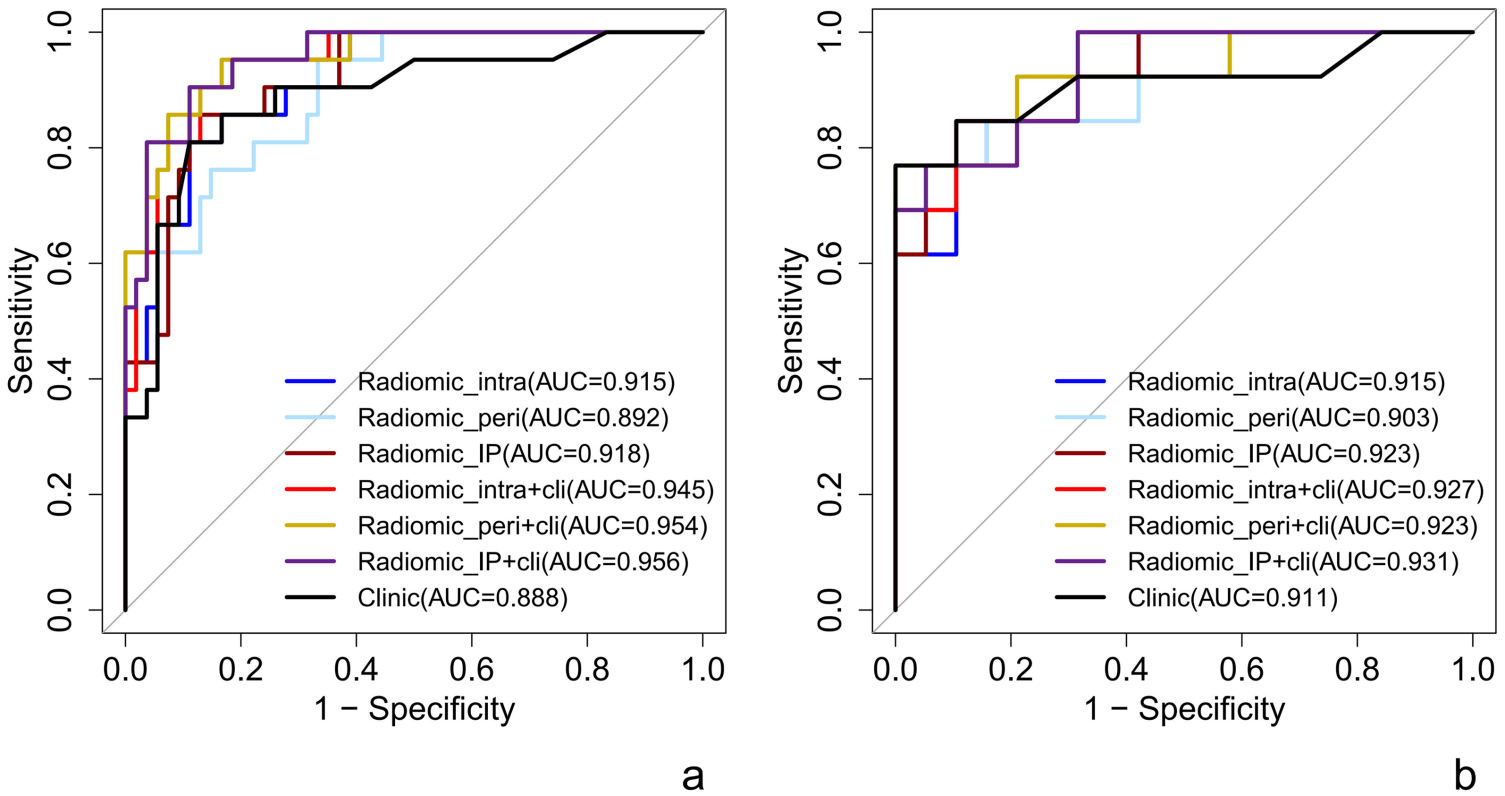
ROC curve of each predictive model in raining set **(a)** and test set **(b)**. Radiomic_intra, intratumoral model; Radiomic_peri, peritumoral model; Radiomic_IP, intra-peritumoral model; Radiomic_intra+clinic, intratumor-clinical model; Radiomic_peri+clinic, peritumor-clinical model; Radiomic_IP + clinic, intra-peri-clinical model.

Among the models evaluated, the intra-peri-clinical model achieved the best performance, with an AUC of 0.96 in the training set and 0.93 in the test set. It exhibited a sensitivity of 0.76 and specificity of 0.96 in the training phase, while in the test set, sensitivity and specificity were 0.91 and 0.86, respectively, with an overall accuracy of 0.89 in training and 0.88 in testing (see Table 3 and Figure 3).

**Table 3:** The performance of each model in the training group and the test group.

Models	AUC		Sensitivity		Specificity		Accuracy	
	training	Test	training	Test	training	Test	training	Test
Intratumor model	0.92	0.92	0.89	0.84	0.81	0.85	0.87	0.84
Peritumor model	0.89	0.90	0.67	1.00	0.95	0.69	0.75	0.88
Clinical model	0.89	0.91	0.74	1.00	0.92	0.86	0.87	0.91
Intra-peritumor model	0.92	0.92	0.89	0.89	0.86	0.85	0.88	0.88
Intratumor-clinical model	0.95	0.93	0.73	1.00	0.96	0.83	0.88	0.88
Peritumor-clinical model	0.95	0.92	0.69	1.00	0.98	0.86	0.87	0.91
Intra-peri-clinical model	0.96	0.93	0.76	0.91	0.96	0.86	0.89	0.88

**Figure 4:**
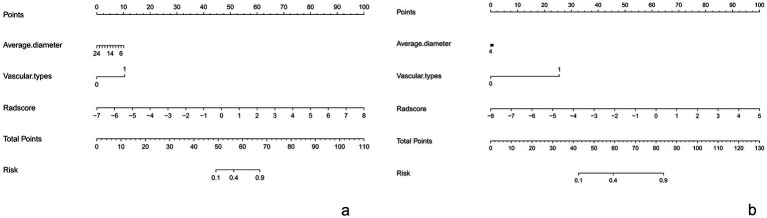
The nomogram of intra-peri-clinical model in training set **(a)** and test set **(b)**.

**Figure 5:**
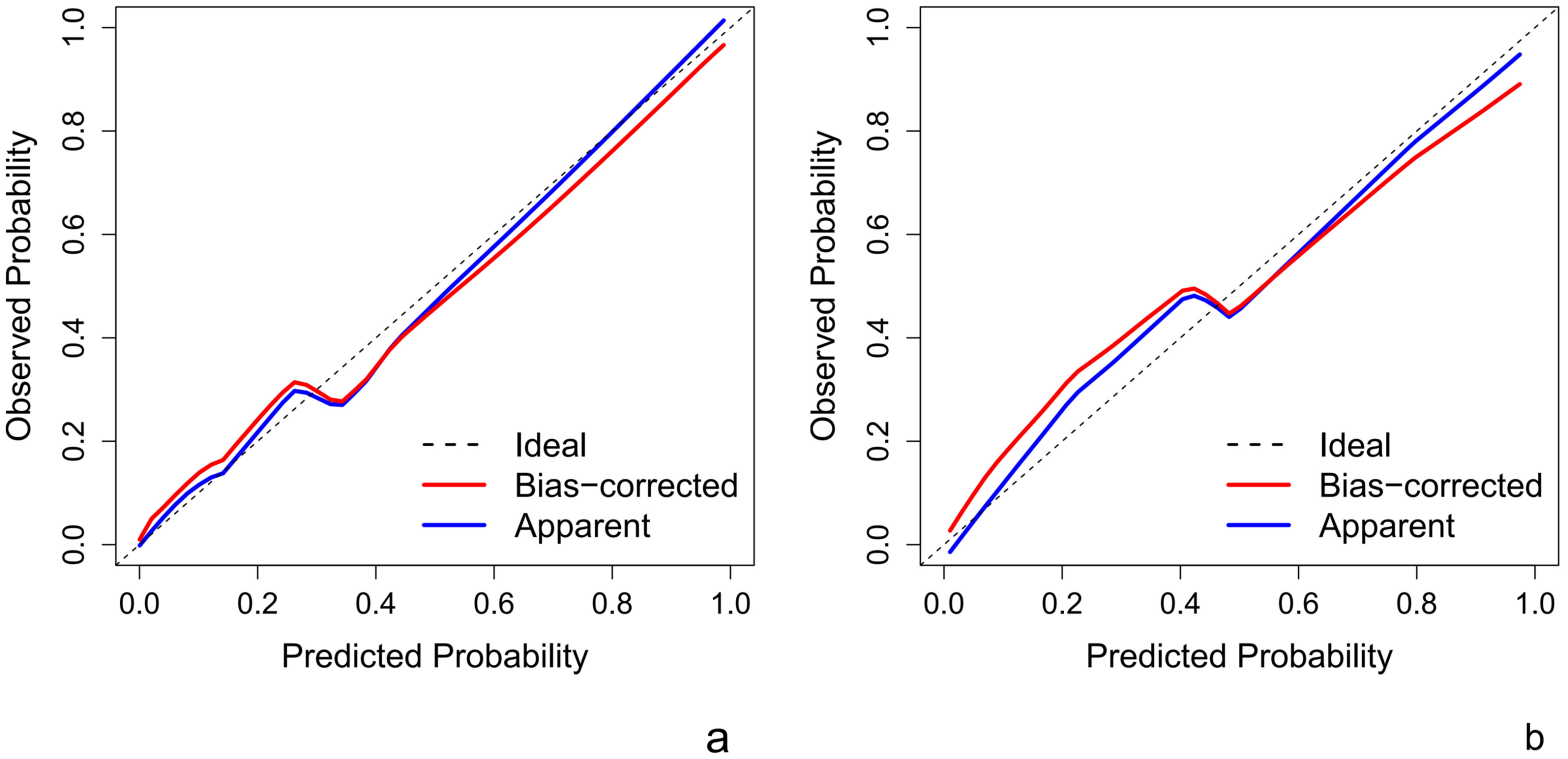
Intra-peri-clinical model calibration curves-training set **(a)** and test sets **(b)**.

**Figure 6:**
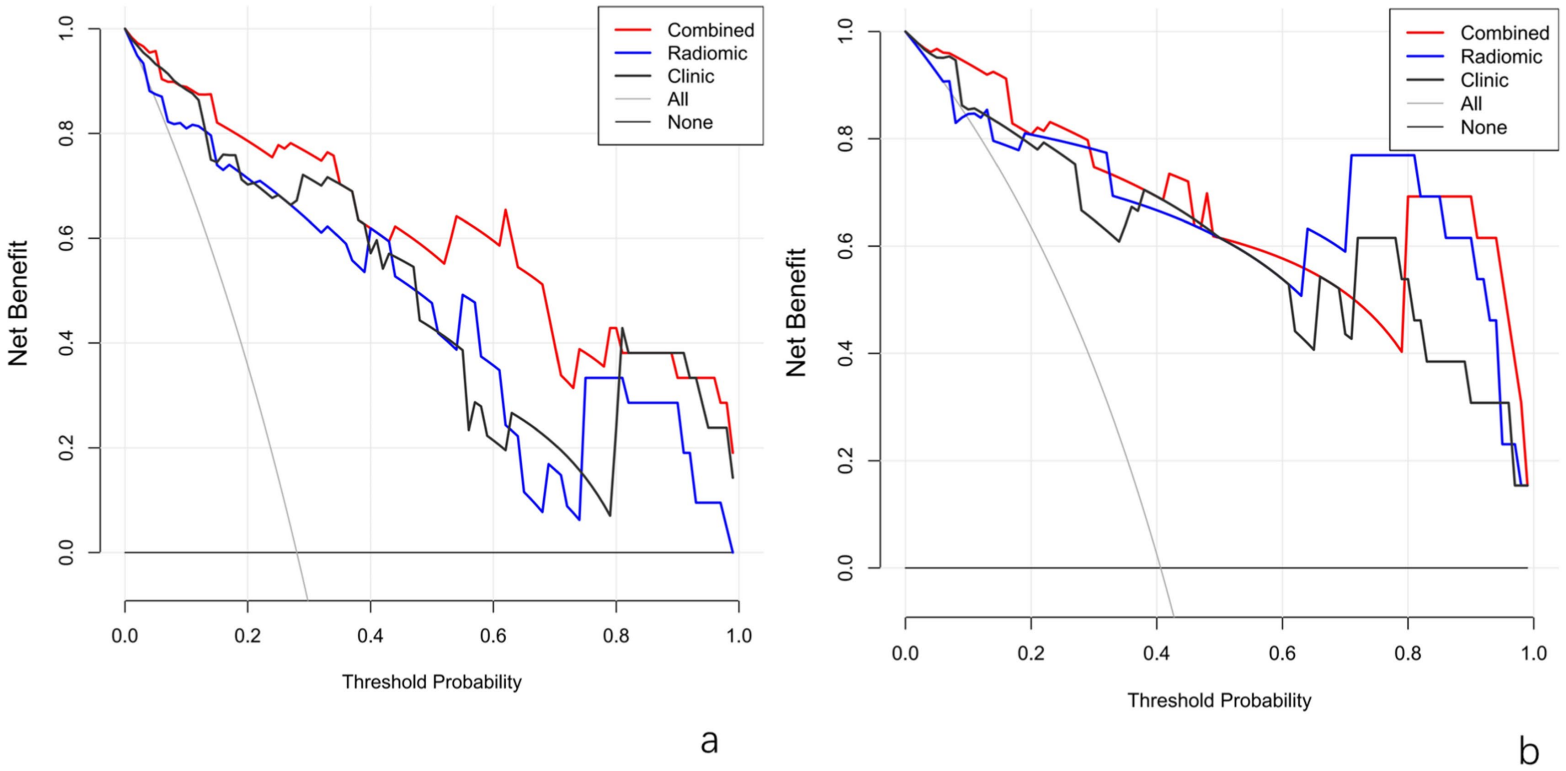
DCA plots of models in the training set **(a)** and test set **(b)**. Radiomic_IP, intra-peritumoral model; Radiomic_IP + clinic, intra-peri-clinical model.

This Nomogram of intra-peri-clinical model enabled the calculation of an overall invasiveness risk score based on factors including the patient’s average nodule diameter, vascular pattern, and intra-peritumoral radiomics scores (Figure 4). The model demonstrated excellent fit across both the training and testing datasets, as evidenced by the well-aligned calibration curves (Figure 5). Furthermore, DCA outcomes highlighted that the intra-peri-clinical model yielded superior clinical net benefits compared with alternative models (Figure 6).

## 4 Discussion

The treatment approach for different pathological types of nodular pulmonary adenocarcinoma varies significantly. AIS and MIA are characterized by slow growth and can remain stable for extended periods. In contrast, IAC often presents with a poor prognosis and rapid infiltration, necessitating prompt surgical intervention. Despite the reliance on preoperative CT scans for diagnosis, subjective factors can influence accuracy. In this study, we explored the potential of modeling to predict the invasiveness of pulmonary adenocarcinoma using a combination of clinical data, CT imaging characteristics, intratumoral and peritumoral radiomics features. Our findings highlighted that the intra-peri-clinical model achieved superior diagnostic performance.

CT scans are crucial for identifying the invasiveness of pulmonary adenocarcinoma, emphasizing morphological and quantitative features. The study identified significant differences between the invasive and non-invasive groups regarding age, gender, nodule types, the location of the lesions, the average diameter of nodules, the shape of the nodule, nodule edge, nodule boundary, burr sign, pleural pulling sign, emphysema background, air bronchus sign, air bubble sign, vascular type, were statistically significant according to Table 1 (all *p* < 0.05). In contrast, no differences were found in the lobulation sign (*p* > 0.05).

Females were more prevalent in both groups, and the invasive group had an older average age. However, some studies suggest that gender differences are not statistically significant, indicating gender has minimal impact on the disease (9, 18). Gu et al. found significant differences in the proportion of lobulation signs between groups (18–20), which is inconsistent with our findings; other imaging features align with our results. In our study, the lobulation sign showed no significant difference among pulmonary nodule subgroups with varying invasiveness, whereas Gu et al. (18) reported statistically significant differences. This discrepancy suggests that the lobulation sign may not be a decisive discriminative feature [as implied by Wang et al. (19), who did not prioritize it] or that its diagnostic value is context-dependent [consistent with the Fleischner guidelines’ emphasis on multiparametric assessment (20)]. Differences between some study results and previous research might be due to single-center research, small sample sizes, and lack of further classification of nodule types, introducing some selection bias.

The clinical model in this study indicated that average nodule diameter and vascular type are independent risk factors for the invasiveness of pulmonary adenocarcinoma (*p* = 0.025; 0.002), which had an AUC of 0.891, with a 95% confidence interval (CI) of 0.816–0.956, sensitivity of 0.706, and specificity of 0.808, and the cutoff value for average nodule diameter is 9.75 mm. Previously, Bu et al. found that the long diameter of pure ground-glass nodules (pGGN) could significantly predict the invasiveness of pulmonary adenocarcinoma (*p* = 0.001), with a cutoff value of 12.5 mm (21). Other studies have found that when the long diameter of ground-glass nodules exceeds 15.37 mm, it usually indicates invasive pulmonary adenocarcinoma (*p* < 0.001), with an AUC of 0.886 for predictive accuracy (22). Hang and Wu’s study showed that the long diameter of the maximum section could independently predict the invasiveness of pulmonary adenocarcinoma (OR = 1.275, *p* < 0.001), with a cutoff value of 11.545 mm and an AUC of 0.776 (23). Zhu et al. found that the short and long diameters of pulmonary nodules had optimal diagnostic values of 9.06 mm and 11.14 mm for predicting invasive pulmonary adenocarcinoma (24). Our cutoff value for nodule diameter differed from previous studies because we calculated the average of the long and short diameters of the nodule. Furthermore, this study included pure ground-glass nodules, partially solid nodules, and solid nodules with diameters not exceeding 30 mm, without separate analysis of cutoff values by nodule type, differing from previous research methods.

Additionally, this study showed that high-grade vascular types are more common in the invasive group, while low-grade vascular types predominate in the non-invasive group, consistent with previous research findings (25–27). The growth and proliferation status of tumors may be closely related to tumor angiogenesis; the higher the malignancy, the richer the blood supply and the more complex the vascular aggregation, which might be one of the mechanisms.

Radiomics research has made significant progress and achievements in various fields (28–30). Weng et al. demonstrated that radiomics has higher differentiation efficiency than traditional CT morphology in distinguishing IAC from MIA (31). Qiu et al. found that the diagnostic efficiency of conventional CT morphology, radiomics, and their combined model is comparable (32). Heng et al. studied the radiomics model of 182 pulmonary adenocarcinoma cases and found that peritumoral radiomics features are extremely important in predicting the pathological classification of pGGN pulmonary adenocarcinoma. Combining intratumor radiomics and clinical factors significantly improved diagnostic performance (training set AUC was 0.958; validation set AUC was 0.895), similar to our results, with good clinical application value (33).

Due to its high invasiveness, pulmonary adenocarcinoma can invade surrounding small blood vessels, lymphatic vessels, and bronchioles, destroying their normal structure and leading to pathological changes such as tumor microvascular formation and bronchiolar obstruction (34). Therefore, the peritumor microenvironment plays a crucial role in disease prediction. Micropathological studies have shown that the average width of the peritumor transition zone in pulmonary adenocarcinoma is about 3.5 mm (35). Including too much normal lung tissue in the analysis can dilute the representativeness of the peritumor microenvironment, thereby reducing predictive accuracy. Based on this, this study extended the edge of the lesion outward by 3 mm as the peritumor ROI to improve predictive performance.

For lung adenocarcinoma, the 3 mm peri-tumoral margin provided the highest AUC (0.94) in malignancy discrimination, compared to 0.85 for 5 mm margins (36). Heng et al. divided patients with pure ground-glass nodular pulmonary adenocarcinoma into IAC and non-IAC groups, sequentially extracted intratumor radiomics features and peritumor 5 mm radiomics features for modeling, and found that the combined model reached an AUC of 0.85 in the validation set (33). Our study constructed models based on intratumor, peritumor 3 mm, and their combined radiomics features, selecting the combined intratumor and peritumor radiomics model with better diagnostic performance. The model was then integrated with independent factors related to CT imaging features, further improving diagnostic performance (both training and test set AUCs were 0.96). DCA showed that the combined model has greater clinical net benefit in identifying the invasiveness of pulmonary adenocarcinoma compared to the clinical model and traditional radiomics models. While our study focuses on CT-based radiomics, PET/CT remains a valuable tool for metabolic characterization of lung nodules (37). The proposed model could be integrated into preoperative planning workflows by combining automated radiomics analysis with clinical parameters in diagnostic software. For instance, radiologists could input nodule diameter, vascular type, and CT images into a tool that generates an invasiveness risk score, guiding surgical decisions (e.g., sublobar vs. lobar resection). Future work should focus on developing user-friendly interfaces and validating real-world clinical utility.

This study still has the following limitations: (1) As a retrospective study, selection bias was unavoidable; (2) This study was a single-center study. Future research should consider including multi-center and larger sample data to demonstrate the generalization ability of the comprehensive model. While our model demonstrated strong performance in internal validation, future multi-center studies with external datasets are necessary to confirm generalizability.

In summary, the combined model based on CT imaging features and CT radiomics features had high predictive performance for the invasiveness of pulmonary nodules, facilitating accurate preoperative assessment of the invasiveness of pulmonary nodules, benefiting patients.

## Data Availability

The raw data supporting the conclusions of this article will be made available by the authors, without undue reservation.

## References

[1] Sung H Ferlay J Siegel RL Laversanne M Soerjomataram I Jemal A. Global Cancer statistics 2020: GLOBOCAN estimates of incidence and mortality worldwide for 36 cancers in 185 countries. CA Cancer J Clin. (2021) 71:209–49. doi: 10.3322/caac.21660, PMID: 33538338

[2] de Koning HJ van der Aalst CM de Jong PA Scholten ET Nackaerts K Heuvelmans MA. Reduced lung-cancer mortality with volume CT screening in a randomized trial. N Engl J Med. (2020) 382:503–13. doi: 10.1056/NEJMoa1911793, PMID: 31995683

[3] Nicholson AG Tsao MS Beasley MB Borczuk AC Brambilla E Cooper WA. The 2021 WHO classification of lung tumors: impact of advances since 2015. J Thorac Oncol. (2022) 17:362–87. doi: 10.1016/j.jtho.2021.11.003, PMID: 34808341

[4] Mazzone PJ Lam L. Evaluating the patient with a pulmonary nodule: a review. JAMA. (2022) 327:264–73. doi: 10.1001/jama.2021.24287, PMID: 35040882

[5] Travis WD Dacic S Wistuba I Nicholson AG Yatabe Y Buettner H. Intraoperative frozen section analysis for guiding limited resection in small-sized lung adenocarcinoma: a multicenter prospective trial. J Clin Oncol. (2021) 39:3283–92. doi: 10.1200/JCO.20.03514, PMID: 34406822 PMC8500586

[6] Ge YH Peng ZL Hu GF. The value of CT diagnostic models in differentiating invasive adenocarcinoma/non-invasive adenocarcinoma. J Pract Radiol. (2021) 37:1970–3.

[7] Guo X Jia X Zhang D Li Y Wang T Chen Z. Indeterminate pulmonary sub-solid nodules in patients with no history of cancer: growing prediction, CT pattern, and pathological diagnosis. Diagn Interv Radiol. (2022) 28:230–8. doi: 10.5152/dir.2022.211100, PMID: 35748205 PMC9634916

[8] Su Y Tao J Lan X Liang C Huang X Zhang J. CT-based intratumoral and peritumoral radiomics nomogram to predict spread through air spaces in lung adenocarcinoma with diameter ≤ 3 cm: a multicenter study. Eur J Radiol Open. (2023) 14:100630. doi: 10.1016/j.ejro.2024.100630, PMID: 39850145 PMC11754163

[9] Dong H Xi Y Liu K Chen L Li Y Pan X. A radiological-Radiomics model for differentiation between minimally invasive adenocarcinoma and invasive adenocarcinoma less than or equal to 3 cm: a two-center retrospective study. Eur J Radiol. (2023) 176:111532. doi: 10.1016/j.ejrad.2024.111532, PMID: 38820952

[10] Lambin P Leijenaar RTH Deist TM Peerlings J Walsh S Caldas C. Radiomics and artificial intelligence in oncology: from data to clinical practice. Nat Rev Clin Oncol. (2022) 19:679–80. doi: 10.1038/s41571-022-00677-3, PMID: 36042382

[11] Zeng Y Chen J Lin S Liu H Zhou Y Zhou X. Radiomics integration based on intratumoral and peritumoral computed tomography improves the diagnostic efficiency of invasiveness in patients with pure ground-glass nodules: a machine learning, cross-sectional, bicentric study. J Cardiothorac Surg. (2023) 20:122. doi: 10.1186/s13019-024-03289-3, PMID: 39934813 PMC11816996

[12] Lv Y Ye J Ling J. Texture analysis to evaluate the invasiveness of lung adenocarcinoma with ground-glass nodule appearance: a comparative study based on non-enhanced and enhanced CT images. Radiol Pract. (2021) 36:1503–8.

[13] Wang C Shao J Lv J Cao Y Zhu C Li J. Deep learning for predicting subtype classification and survival of lung adenocarcinoma on computed tomography. Transl Oncol. (2021) 14:101141. doi: 10.1016/j.tranon.2021.101141, PMID: 34087705 PMC8184655

[14] Park S Lee SM Noh HN Hwang HJ Kim S do KH. Differentiation of predominant subtypes of lung adenocarcinoma using a quantitative radiomics approach on CT. Eur Radiol. (2020) 30:4883–92. doi: 10.1007/s00330-020-06805-w, PMID: 32300970

[15] Liang B Tong C Nong J Zhang Y. Histological subtype classification of non-small cell lung cancer with radiomics and 3D convolutional. Neural Netw. (2023) 37:2895–909. doi: 10.1007/s10278-024-01152-4, PMID: 38861072 PMC11612112

[16] Shao X Niu R Shao X Jiang Z Wang Y. Value of 18F-FDG PET/CT-based radiomics model to distinguish the growth patterns of early invasive lung adenocarcinoma manifesting as ground-glass opacity nodules. EJNMMI Res. (2020) 10:80. doi: 10.1186/s13550-020-00668-4, PMID: 32661639 PMC7359213

[17] Zhang B Qi S Pan X Li C Yao Y Qian W. Deep CNN model using CT radiomics feature mapping recognizes EGFR gene mutation status of lung adenocarcinoma. Front. Oncologia. (2020) 10:10 598721. doi: 10.3389/fonc.2020.598721, PMID: 33643902 PMC7907520

[18] Gu XL Liu Z Shao WP Feng HX Zhang ZR Sun HL. Predictive value of CT features for pathological subtypes of lung nodules. Chin J Thor Cardiov Surg. (2022) 29:684–92. doi: 10.3760/cma.j.issn.1001-9324.2022.07.010

[19] Wang H Weeng Q Hui J Fang S Wu X Mao W. Value of TSCT features for differentiating preinvasive and minimally invasive adenocarcinoma from invasive adenocarcinoma presenting as subsolid nodules smaller than 3 cm. Acad Radiol. (2020) 27:395–403. doi: 10.1016/j.acra.2019.05.005, PMID: 31201034

[20] MacMahon H Naidich DP Goo JM Lee KS Leung ANC Mayo RJ. Guidelines for Management of Incidental Pulmonary Nodules: Common Questions and Challenging Scenarios. Radiology. (2023) 306:e230511. doi: 10.1148/radiol.230511

[21] Bu YL Li Y Qi YG Liu LH. Study on the correlation between high-resolution CT signs and pathological histology of pure ground-glass density nodules. J Clin Radiol. (2018) 37:247–50. doi: 10.13437/j.cnki.jcr.2018.02.016

[22] Hu XQ Huang YY Wan B Liu SB. Value of CT gray histogram in differentiating the pathological subtypes of adenocarcinoma in sub-20 mm subsolid nodules. J Clin Radiol. (2021) 40:712–6. doi: 10.13437/j.cnki.jcr.2021.04.020

[23] Hang YQ Wu XH. Research on the prediction of invasiveness in ground-glass nodule adenocarcinoma by CT radiomics. J Clin Radiol. (2024) 43:356–61. doi: 10.13437/j.cnki.jcr.2024.03.009

[24] Vermilion p Ma JL Wang H Liu F. Correlation analysis between artificial intelligence quantitative lung nodule parameters and the degree of lung adenocarcinoma invasion. J Clin Pulmon Med. (2024) 29:7-10+17. doi: 10.3969/j.issn.1009-6663.2024.01.002

[25] Guo CR Han R Xue F Xu L Ren WG Li M. Expression and clinical significance of CD31, CD34, and CD105 in pulmonary ground glass nodules with different vascular manifestations on CT. Front Oncol. (2022) 12:956451. doi: 10.3389/fonc.2022.956451, PMID: 36185269 PMC9521677

[26] Lee HY Choi YL Lee KS Han J Zo JI Shim YM. Pure ground-glass opacity neoplastic lung nodules: histopathology, imaging, and management. Am J Roentgenol. (2014) 202:W224–33. doi: 10.2214/AJR.13.11819, PMID: 24555618

[27] Zhang C Li J Sun M Li S Li J Li Q. Peripheral vessel and air bronchograms for detecting the pathologic patterns of subsolid nodules. Clin Imaging. (2019) 56:63–8. doi: 10.1016/j.clinimag.2019.03.010, PMID: 30933847

[28] Zhang PJ Li TR Tao XM Jin X Zhao SH. Analysis of CT features of ground-glass nodules with early lepidic growth predominant invasive adenocarcinoma and other pathological subtypes. Chin J Radiol. (2021) 55:739–44.

[29] Huang ZC Ye DL Hu JS Li XY Lv WH Zhou CS. Distinguishing small cell lung cancer from non-small cell lung cancer based on a CT radiomics model. Chin J Int Imag Ther. (2021) 18:474–8.

[30] Hu K Zhang X Bai BS Wang ZJ Cai Q Liu YL. CT radiomics model predicts prognosis in patients with EGFR-mutated non-small cell lung cancer treated with targeted therapy. Chin J Med Imag Technol. (2022) 38:1491–5. doi: 10.13929/j.issn.1003-3289.2022.10.011

[31] Weng Q Zhou L Wang H Hui J Chen M Pang P. A radiomics model for determining the invasiveness of solitary pulmonary nodules that manifest as part-solid nodules. Clin Radiol. (2019) 74:933–43. doi: 10.1016/j.crad.2019.07.026, PMID: 31521324

[32] Qiu L Zhang X Mao H Fang X Ding W Zhao L. Comparison of comprehensive morphological and radiomics features of subsolid pulmonary nodules to distinguish minimally invasive adenocarcinomas and invasive adenocarcinomas in CT scan. Front Oncol. (2022) 11:69112. doi: 10.3389/fonc.2021.691112, PMID: 35059308 PMC8765579

[33] Zhang H Liu JB Yu YM Li ZT Wang M. Discussion on the predictive value of combined intra-and peri-tumoral radiomics and clinical factors for pathological typing of pure ground-glass nodules. Chin J Cancer Prevent Treat. (2022) 29:508-515, 522. doi: 10.16073/j.cnki.cjcpt.2022.07.10

[34] Altorki NK Markowitz GJ Gao D Port JL Saxena A Stiles B. The lung microenvironment: an important regulator of tumor growth and metastasis. Nat Rev Cancer. (2019) 19:9–31. doi: 10.1038/s41568-018-0081-9, PMID: 30532012 PMC6749995

[35] Zhang L Yankelevitz DF Henschke CI Jirapatnakul AC Reeves AP Carter D. The peritumoral stromal width in lung adenocarcinoma: a histopathological correlate of CT-based radiomic features. J Thorac Oncol. (2022) 17:718–23. doi: 10.1016/j.jtho.2022.01.012 35181499

[36] Lambin P Rios-Velazquez E Leijenaar R. Radiomics of the peritumoral transition zone: a universal biomarker for solid tumor prognosis. Nat Commun. (2021) 12:4887. doi: 10.1038/s41467-021-25182-6, PMID: 34373446

[37] Evangelista L Cuocolo A Pace L Mansi L del Vecchio S Miletto P. Performance of FDG-PET/CT in solitary pulmonary nodule based on pre-test likelihood of malignancy: results from the ITALIAN retrospective multicenter trial. Eur J Nucl Med Mol Imaging. (2018) 45:1898–907. doi: 10.1007/s00259-018-4016-1, PMID: 29736699

